# Upregulation of *CKIP*-*1* inhibits high-glucose induced inflammation and oxidative stress in HRECs and attenuates diabetic retinopathy by modulating *Nrf2*/*ARE* signaling pathway: an in vitro study

**DOI:** 10.1186/s13578-019-0331-x

**Published:** 2019-08-23

**Authors:** Lan Zhang, Jie Yu, Mingxia Ye, Hailan Zhao

**Affiliations:** 1 Department of Ophthalmology, Zhejiang Provincial People’s Hospital, No.158, Shangtang Road, Xiacheng District, Hangzhou, 310014 Zhejiang China; 2 Department of Ophthalmology, People’s Hospital of Hangzhou Medical College, No.128, ShangTang Road, XiaCheng District, Hangzhou, 310014 Zhejiang China

**Keywords:** Diabetic retinopathy, Inflammation, Oxidative stress, *CKIP*-*1*, *Nrf2*/*ARE* signal pathway

## Abstract

**Purpose:**

The aim of this study was to investigate the underlying mechanisms of diabetic retinopathy (DR) development.

**Methods:**

Real-Time qPCR was used to detect *Casein kinase 2 interacting protein*-*1* (*CKIP*-*1*) and *Nuclear factor E2*-*related factor 2* (*Nrf2*) mRNA levels. Western Blot was employed to detect protein levels. Malondialdehyde (MDA) assay kit, superoxide dismutase (SOD) kit and glutathione peroxidase (GSH-Px) kit were used to evaluate oxidative stress in high-glucose treated human retinal endothelial cells (HRECs). Calcein-AM/propidium iodide (PI) double stain kit was employed to detect cell apoptosis. Enzyme-linked ImmunoSorbent Assay (ELISA) was used to detect inflammation associated cytokines secretion. Co-immunoprecipitation (CO-IP) was performed to investigate the interactions between *CKIP*-*1* and *Nrf2*. Luciferase reporter gene system was used to detect the transcriptional activity of *Nrf2*.

**Results:**

*CKIP*-*1* was significantly downregulated in either DR tissues or high-glucose treated HRECs comparing to the Control groups. Besides, high-glucose (25 mM) inhibited HRECs viability and induced oxidative stress, inflammation associated cytokines (TNF-α, IL-6 and IL-1β) secretion and cell apoptosis, which were all reversed by synergistically overexpressing *CKIP*-*1* and aggravated by knocking down *CKIP*-*1*. Of note, we found that overexpressed *CKIP*-*1* activated *Nrf2*/*ARE* signaling pathway and increased its downstream targets including *HO*-*1*, *NQO*-*1*, *γGCS* and *SOD* in high-glucose treated HRECs. Further results also showed that *CKIP*-*1* regulated cell viability, oxidative stress, inflammation and apoptosis in high-glucose treated HRECs by activating *Nrf2*/*ARE* signaling pathway.

**Conclusion:**

We concluded that overexpressed *CKIP*-*1* alleviated DR progression by activating *Nrf2*/*ARE* signaling pathway.

## Introduction

Diabetic retinopathy (DR) is a common complication of type 1 or type 2 diabetes mellitus (DM) [1], however, the underlying mechanisms are still not fully delineated. Researchers found that 721 candidate genes might involve in DR progression [2]. In addition, researchers found that microRNA-183 [3] and miR-451a [4] also regulated DR progression. Besides, oxidative stress [5] and inflammation [6] were also pivotal for DR pathogenesis. For example, attenuation of oxidative stress by Gabapentin could help to alleviate DR in rats [7] and anti-inflammation drugs treatment attenuated DR in streptozotocin (STZ)-induced diabetic rats [8]. Hence targeting oxidative stress and inflammation might help to cure DR in clinic.

*Casein kinase 2 interacting protein*-*1* (*CKIP*-*1*) participated in the regulation of multiple cell functions, such as cell proliferation [9, 10], apoptosis [11], inflammation [12, 13] and oxidative stress [11]. For example, *CKIP*-*1* regulated the proliferative abilities of non-Hodgkin’s lymphoma cells [10] and macrophages [9]. Besides, *CKIP*-*1* also influenced cell apoptosis and oxidative stress, specifically, *CKIP*-*1* alleviated oxygen–glucose deprivation/reoxygenation induced and oxidative stress in hippocampal neurons [11]. In addition, *CKIP*-*1* could regulate immune system, and CKIP-1 modulated inflammatory reactions by regulating M1 and M2 inflammatory macrophage polarization [13]. Since oxidative stress and inflammation are two important characteristics of DR, it is reasonable to speculate that CKIP-1 might be crucial for DR progression by regulating inflammation and oxidative stress. Notably, our preliminary experiments showed that *CKIP*-*1* was aberrantly low-expressed in DR tissues and high-glucose treated HRECs comparing to the Control groups. The above results indicated that *CKIP*-*1* might be the hub gene in DR progression by regulating oxidative stress and inflammation, and targeting *CKIP*-*1* will provide new therapeutic agent for DR treatment.

*Nuclear factor E2*-*related factor 2* (*Nrf2*)/*antioxidant response element* (*ARE*) pathway is the key regulator of oxidative stress [14], inflammation [15], cell proliferation [16] and apoptosis [17], and *Nrf2*/*ARE* signaling pathway was proved to be the downstream target of *CKIP*-*1* in cultured hippocampal neurons [11]. Interestingly, *CKIP*-*1* ameliorated high-glucose induced expression of fibronectin and *intercellular cell adhesion molecule*-*1* (*ICAM*-*1*) in glomerular mesangial cells by activating *Nrf2*/*ARE* signaling pathway [18]. In addition, *Nrf2*/*ARE* signaling pathway involved in the regulation of the cell damages caused by high-glucose treatment [19, 20]. Therefore, the *Nrf2*/*ARE* signaling pathway might be the downstream target of *CKIP*-*1* in DR development, however, the mechanisms are still unclear.

Taken together, we hypothesized that *CKIP*-*1* might regulate DR progression by modulating *Nrf2*/*ARE* signaling pathway mediated cell proliferation, apoptosis, oxidative stress and inflammation. This study will uncover the underlying mechanisms of DR progression regulated by *CKIP*-*1*, and provide new therapeutic agents for DR treatment in clinic.

## Results

### The expression levels of *CKIP*-*1* in DR tissues and high-glucose treated HRECs

**Fig. 1:**
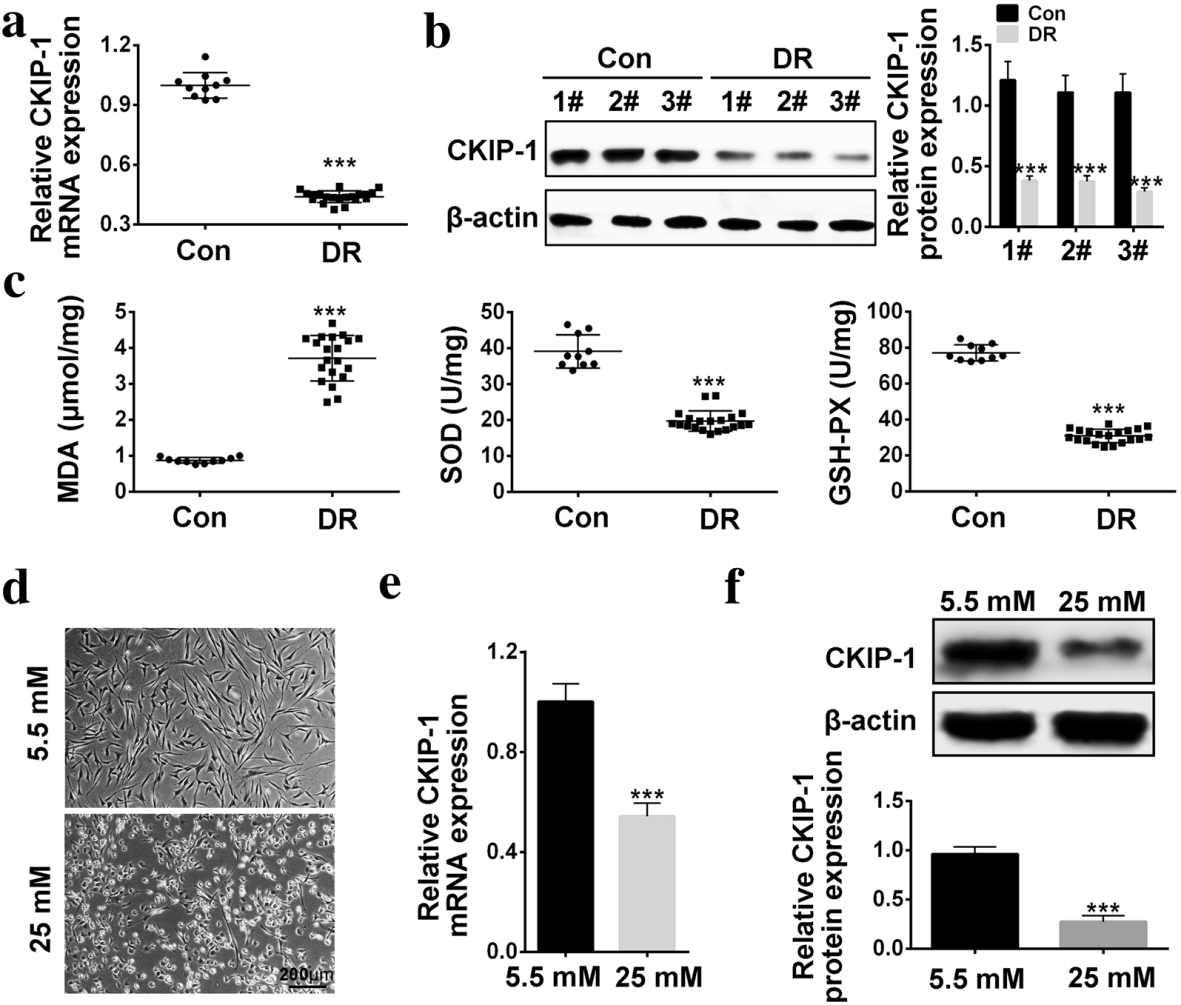
*CKIP*-*1* was downregulated in DR tissues and high-glucose treated HRECs. **a** Real-Time qPCR was used to detect *CKIP*-*1* mRNA levels in DR tissues (N = 20) and normal tissues (N = 10). **b** Western Blot Was performed to detect *CKIP*-*1* protein levels in DR tissues (The “1#, 2# and 3#” represented 3 individual clinical specimens). **c** MDA assay kit was used to detect MDA levels, SOD kit was used to detect SOD activity and GSH-Px kit was used to detect GSH-Px activity in DR tissues respectively (n = 3). **d** The morphology of HRECs treated with low-glucose (5.5 mM) and high-glucose (25 mM) (Scale bar is 200 μm). **e** Relative *CKIP*-*1* mRNA levels in HRECs treated with low-glucose (5.5 mM) and high-glucose (25 mM) was detected by Real-Time qPCR (n = 3). **f** Relative *CKIP*-*1* protein levels in HRECs treated with low-glucose (5.5 mM) and high-glucose (25 mM) was detected by Western Blot (The “1#, 2# and 3#” represented 3 individual repetitions). The data above in one experiments were repeated at least 3 times and performed as mean ± standard deviation (SD), *P < 0.05, **P < 0.01 and ***P < 0.001

*CKIP*-*1* was reported to be closely related with high-glucose induced diabetic nephropathy (DN) [18], hence we speculated that *CKIP*-*1* might also participate in the development of DR. To validate the hypothesis, *CKIP*-*1* expression levels were first evaluated in the clinical tissues. The results showed that *CKIP*-*1* was downregulated in DR tissues comparing to the normal tissues (Fig. 1a, b). Of note, the results also showed that MDA levels was increased, SOD activity and GSH-PX activity were decreased in DR tissues comparing to the normal tissues (Fig. 1c), which suggested that oxidative stress played an important role in DR progression. In addition, the cellular results was in accordance with the clinical results, high-glucose treatment changed the morphology of HRECs from spindle-shape to round-shape (Fig. 1d), and decreased *CKIP*-*1* levels in HRECs comparing to the control group (Fig. 1e, f). which indicated that cell viability and functions were affected by high-glucose treatment. Furthermore, the results showed that high-glucose (25 mM) inhibited cell viability and promoted inflammation cytokines (TNF-α, IL-6 and IL-1β) secretion in a time dependent manner (Additional file 1: Figure S1).

### Overexpressed *CKIP*-*1* reverses the effects of high-glucose on HRECs viability, oxidative stress, inflammation and apoptosis

**Fig. 2:**
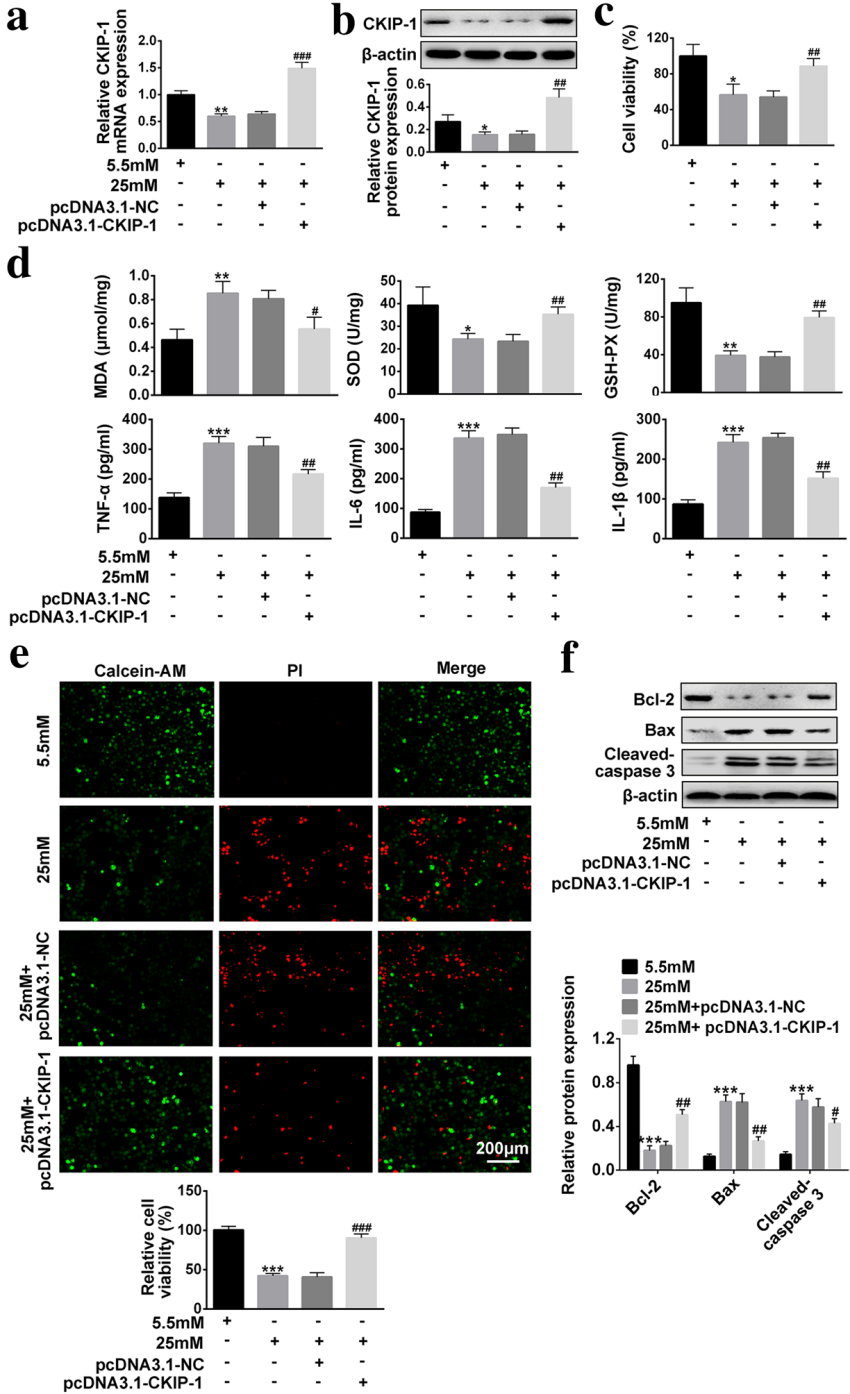
The effects of overexpressed *CKIP*-*1* on high-glucose treated HRECs in terms of cell viability, oxidative stress, inflammation and apoptosis. **a** Relative *CKIP*-*1* mRNA levels were detected by Real-Time qPCR (n = 3). **b** Relative *CKIP*-*1* protein levels were detected by Western Blot (n = 3). **c** CCK-8 assay kit was utilized to detect cell viability (n = 3). **d** MDA levels, SOD activity and GSH-PX activity were detected by MDA assay kit, SOD kit abd GSH-PX kit respectively (n = 3). Inflammation associated cytokines (TNF-α, IL-6 and IL-1β) were detected by ELISA (n = 3). **e** Calcein-AM/PI double stain kit was employed to detect cell apoptosis (Scale bar is 200 μm). **f** Apoptosis associated proteins (*Bcl*-*2*, *Bax* and *Cleaved Caspase 3*) were detected by Western Blot (n = 3). The data above in one experiments were repeated at least 3 times and performed as mean ± standard deviation (SD), *P < 0.05, **P < 0.01 and ***P < 0.001

We next explored the influences of overexpressed *CKIP*-*1* on high-glucose treated HRECs in terms of cell viability, oxidative stress, inflammation and apoptosis. The results showed that high-glucose (25 mM) significantly decreased *CKIP*-*1* levels in HRECs comparing to the low-glucose group (5.5 mM), and pcDNA3.1-*CKIP*-*1* was used to overexpress *CKIP*-*1* in high-glucose (25 mM) treated HRECs (Fig. 2a, b). The CCK-8 results showed that cell viability was inhibited by high-glucose (25 mM) treatment, which was reversed by *CKIP*-*1* overexpression (Fig. 2c). The further results showed that overexpressed *CKIP*-*1* also abrogated the effects of high-glucose (25 mM) on oxidative stress, inflammation and apoptosis (Fig. 2d–f). Specifically, overexpressed *CKIP*-*1* abrogated the effects of high-glucose (25 mM) on MDA levels, SOD activity and GSH-PX activity (Fig. 2d). High-glucose (25 mM) promoted secretions of inflammation associated cytokines (TNF-α, IL-6 and IL-1β), which were abrogated by synergistically overexpressing *CKIP*-*1* (Fig. 2d). The Calcein-AM/PI double stain assay results showed that overexpressed *CKIP*-*1* abrogated the promoting effects of high-glucose (25 mM) on cell apoptosis (Fig. 2e). The Western Blot results showed that high-glucose (25 mM) promoted expressions of pro-apoptotic proteins (*Bax* and *cleaved*-*Caspase 3*) and inhibited anti-apoptotic protein (*Bcl*-*2*), which were reversed by *CKIP*-*1* overexpression (Fig. 2f).

### Knock-down of *CKIP*-*1* aggravated the effects of high-glucose on HRECs viability, oxidative stress, inflammation and apoptosis

**Fig. 3:**
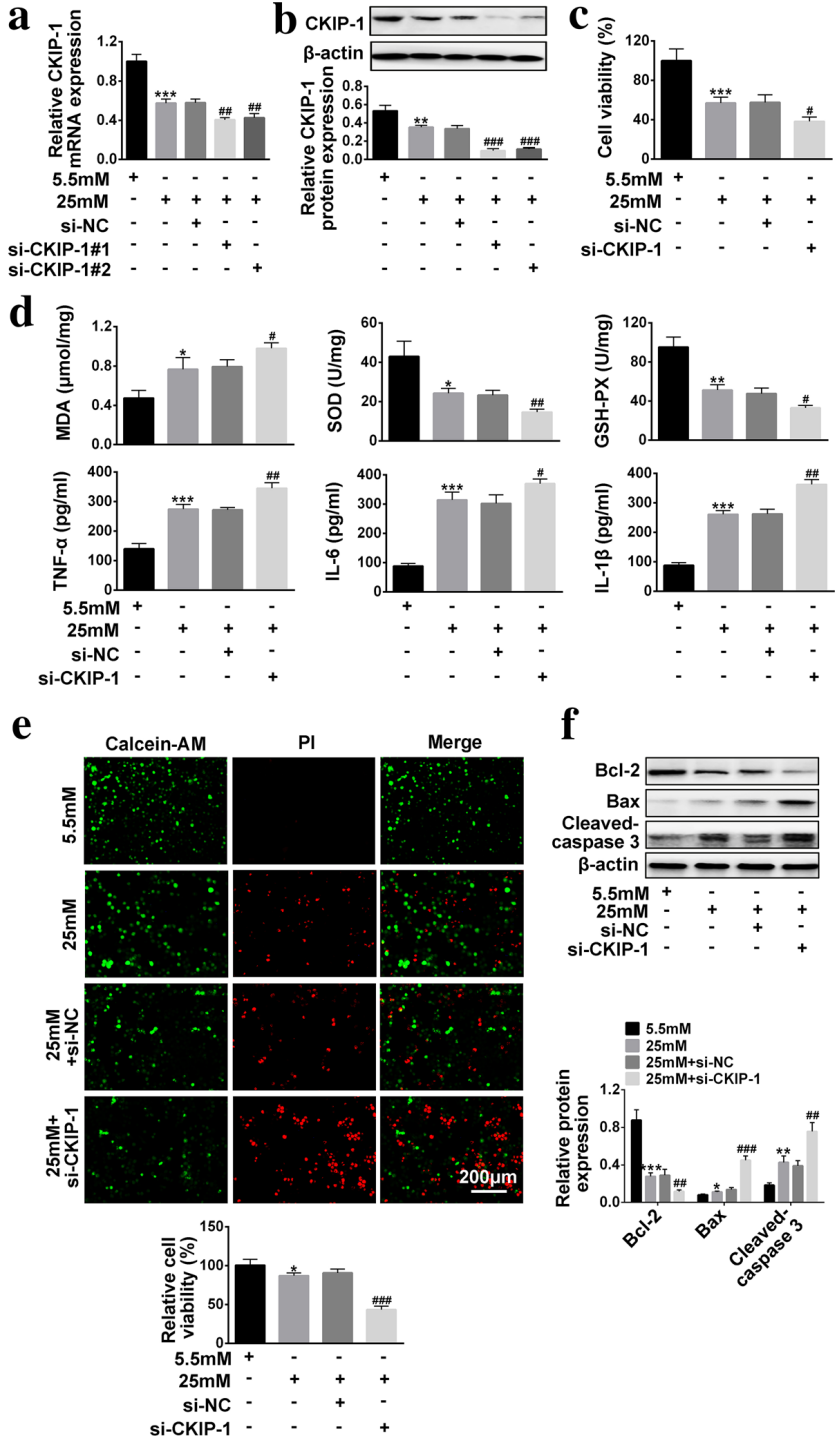
The effects of *CKIP*-*1* knock-down on high-glucose treated HRECs in terms of cell viability, oxidative stress, inflammation and apoptosis. **a** Relative *CKIP*-*1* mRNA levels were detected by Real-Time qPCR (n = 3). **b** Relative *CKIP*-*1* protein levels were detected by Western Blot (n = 3). **c** CCK-8 assay kit was utilized to detect cell viability. **d** MDA levels, SOD activity and GSH-PX activity were detected by MDA assay kit, SOD kit abd GSH-PX kit respectively (n = 3). Inflammation associated cytokines (TNF-α, IL-6 and IL-1β) were detected by ELISA (n = 3). **e** Calcein-AM/PI double stain kit was employed to detect cell apoptosis (Scale bar is 200 μm). **f** Apoptosis associated proteins (*Bcl*-*2*, *Bax* and *Cleaved Caspase 3*) were detected by Western Blot (n = 3). The data above in one experiments were repeated at least 3 times and performed as mean ± standard deviation (SD), *P < 0.05, **P < 0.01 and ***P < 0.001

We next explored the effects of *CKIP*-*1* knock-down on high-glucose regulated HRECs viability, oxidative stress, inflammation and apoptosis. The results showed that we successfully established *CKIP*-*1* knock-down HRECs (Fig. 3a, b). High-glucose treatment inhibited HRECs viability, which was aggravated by synergistically downregulating *CKIP*-*1* (Fig. 3c). Similarly, knock-down of *CKIP*-*1* enhanced the effects of high-glucose on SOD activity, GSH-PX activity and MDA levels (Fig. 3d), which indicated that synergistically knock-down of *CKIP*-*1* aggravated the promoting effects of high-glucose treatment on HRECs oxidative stress. Aside from cell viability and oxidative stress, knock-down of *CKIP*-*1* also enhanced the effects of high-glucose treatment on inflammation associated cytokines (TNF-α, IL-6 and IL-1β) secretion (Fig. 3d) and cell apoptosis (Fig. 3e, f).

### The effects of overexpressed *CKIP*-*1* on *Nrf2*/*ARE* signaling pathway activation in high-glucose treated HRECs

**Fig. 4:**
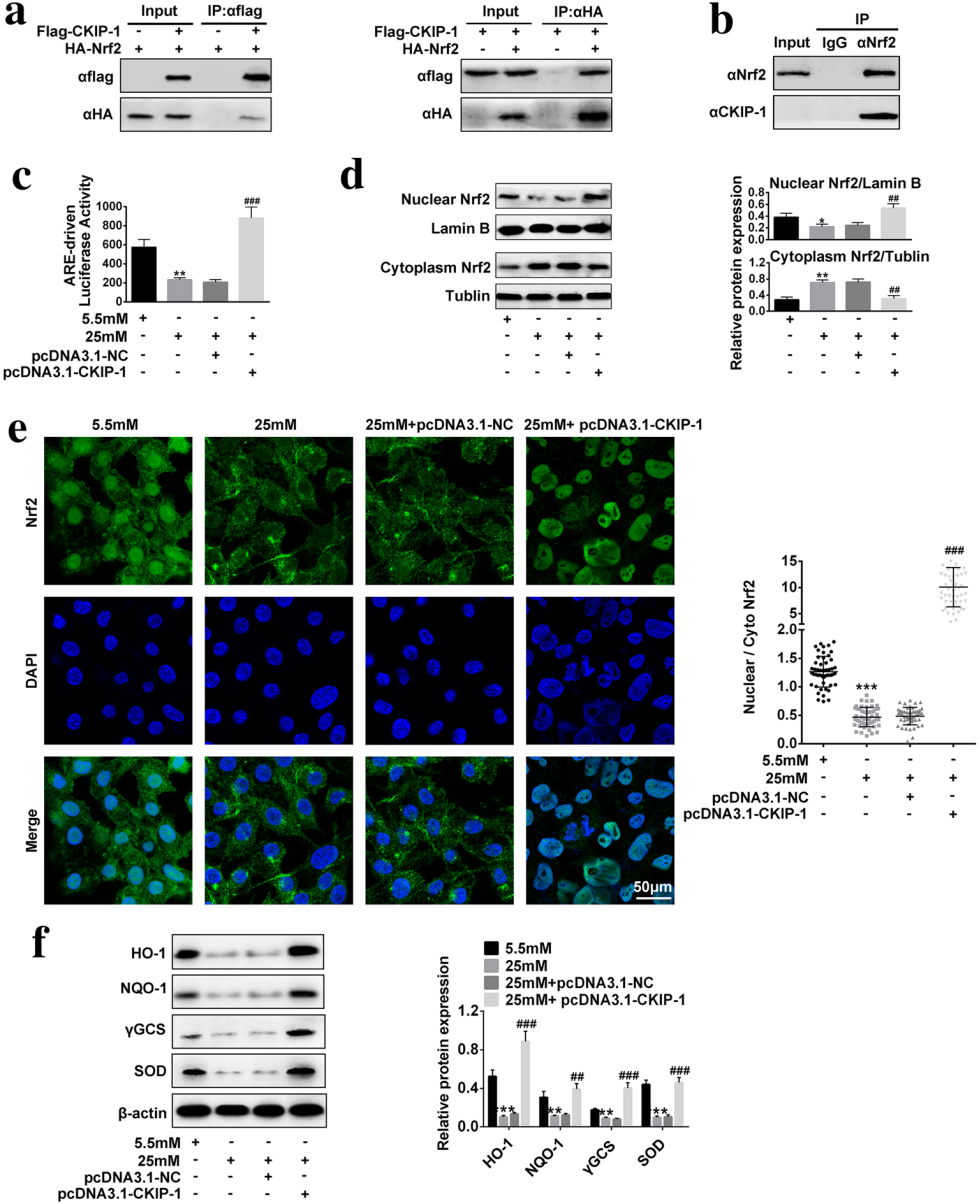
*Nrf2*/*ARE* signaling pathway was activated by *CKIP*-*1* in high-glucose treated HRECs. **a**, **b** Co-IP was used to investigate the protein–protein interactions of *CKIP*-*1* and *Nrf2* (n = 3). **c** Luciferase reporter gene system was used to detect the transcriptional activity of *Nrf2* (n = 3). **d** Western Blot was used to detect nuclear *Nrf2* and cytoplasm *Nrf2* expressions (n = 3). **e** Immunofluorescence assay was performed to detect the expression levels and cellular localization of *Nrf2* (Scale bar is 50 μm). **f** The downstream targets of *Nrf2* (including *HO*-*1*, *NQO*-*1*, *γGCS* and *SOD*) were detected by Western Blot (n = 3). The data above in one experiments were repeated at least 3 times and performed as mean ± standard deviation (SD), *P < 0.05, **P < 0.01 and ***P < 0.001

It was reported that *Nrf2*/*ARE* signaling pathway is the downstream target of *CKIP*-*1* [21], hence we next explored whether *CKIP*-*1* could regulate *Nrf2*/*ARE* signaling pathway in high-glucose treated HRECs. The CO-IP results showed that *CKIP*-*1* interacted with *Nrf2*, which could further form *CKIP*-*1*–*Nrf2* protein complex in HRECs (Fig. 4a, b). We also found that high-glucose treatment significantly inhibited the transcriptional activity of *Nrf2*, which was reversed by overexpressing *CKIP*-*1* (Fig. 4c). Of note, our results showed that high-glucose treatment affected the cellular localization of *Nrf2* in HRECs (Fig. 4d, e). Specifically, high-glucose treatment increased cytoplasm *Nrf2* levels, but decreased nuclear *Nrf2* levels, which were significantly reversed by *CKIP*-*1* overexpression (Fig. 4d, e). The results indicated that overexpressed *CKIP*-*1* promoted *Nrf2* translocation from cytoplasm to nuclear and facilitating the activation of downstream targets of *Nrf2*. Accordingly, our results proved that high-glucose inhibited the expressions of the downstream targets of *Nrf2* (including *HO*-*1*, *NQO*-*1*, *γGCS* and *SOD*) in HRECs, which were also reversed by synergistically transfecting cells with *CKIP*-*1* overexpression vectors (Fig. 4f).

### *CKIP*-*1* regulated cell viability, oxidative stress, inflammation and apoptosis of high-glucose treated HRECs by activating *Nrf2*/*ARE* signaling pathway

**Fig. 5:**
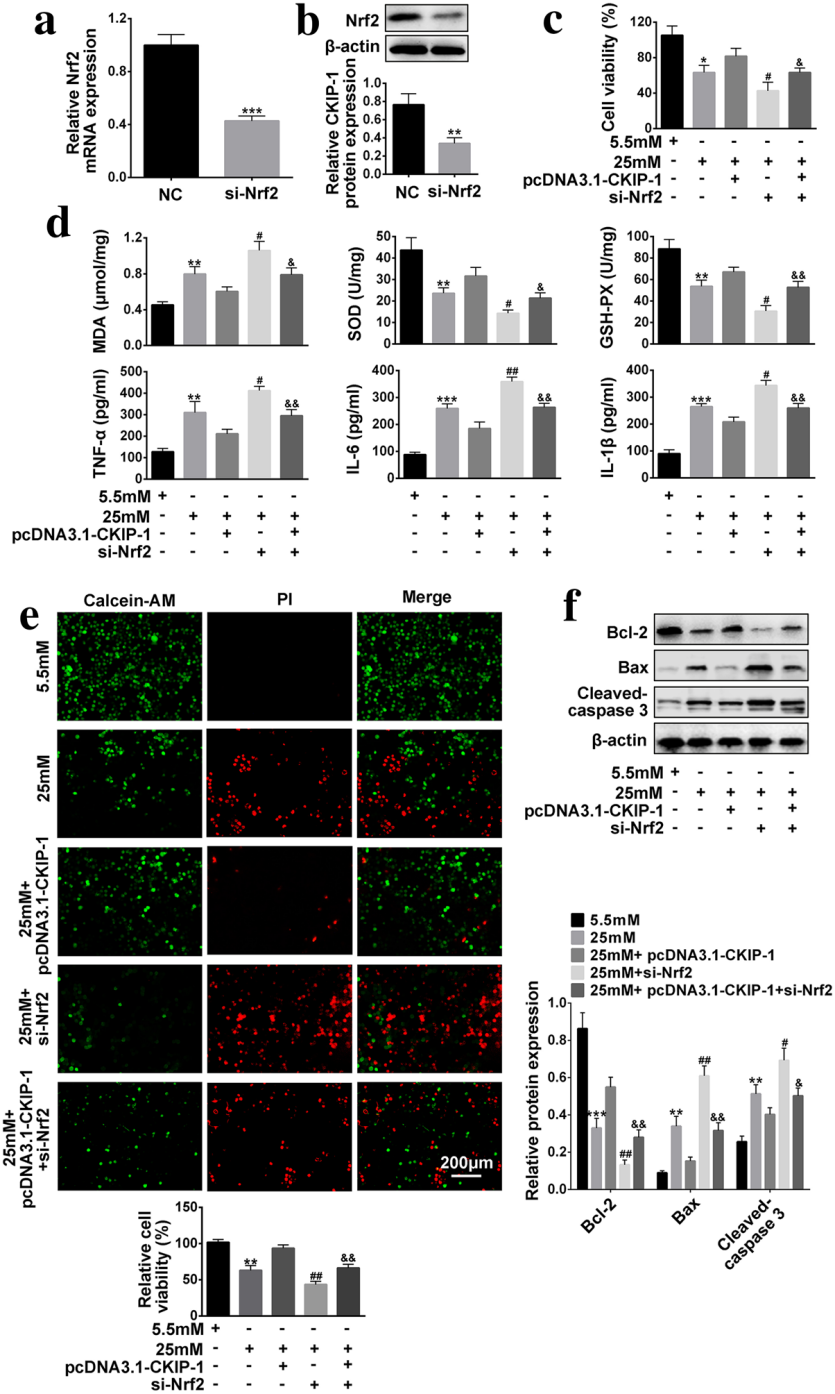
Knock-down of *Nrf2* reverses the effects of overexpressed *CKIP*-*1* on cell viability, oxidative stress, inflammation and apoptosis of high-glucose treated HRECs. **a** Real-Time qPCR was used to detect relative *Nrf2* mRNA levels (n = 3). **b** Western Blot was employed to detect relative *Nrf2* protein expressions (n = 3). **c** Cell viability was evaluated by CCK-8 assay kit (n = 3). **d** MDA levels, SOD activity and GSH-PX activity were detected by MDA assay kit, SOD kit abd GSH-PX kit respectively (n = 3). Inflammation associated cytokines (TNF-α, IL-6 and IL-1β) were detected by ELISA (n = 3). **e** Calcein-AM/PI double stain kit was employed to detect cell apoptosis (Scale bar is 200 μm). **f** Apoptosis associated proteins (*Bcl*-*2*, *Bax* and *Cleaved Caspase 3*) were detected by Western Blot (n = 3). The data above in one experiments were repeated at least 3 times and performed as mean ± standard deviation (SD), *P < 0.05, **P < 0.01 and ***P < 0.001

*Nrf2*/*ARE* signaling pathway has been reported to participate in the regulation of cell viability [21], oxidative stress [22], inflammation [23] and apoptosis [22], which has also been proved to be regulated by *CKIP*-*1* in the previous literature [21] and our experiments. Hence we next explored the roles of *CKIP*-*1* and *Nrf2*/*ARE* signaling pathway in DR progression. We successfully established *Nrf*-*2* knock-down HRECs models (Fig. 5a, b). Further results showed that overexpressed *CKIP*-*1* abolished the inhibiting effects of high-glucose on cell viability, which were reversed by synergistically knocking down *Nrf2* in HRECs (Fig. 5c). In addition to cell viability, *CKIP*-*1* and *Nrf2* also have similar effects on oxidative stress, inflammation and cell apoptosis. Specifically, the effects of overexpressed *CKIP*-*1* on oxidative stress (MDA levels, SOD activity and GSH-PX activity), inflammation associated cytokines (TNF-α, IL-6 and IL-1β) secretions and apoptosis in HRECs treated with high-glucose were reversed by synergistically knocking down *Nrf2* (Fig. 5d–f).

## Discussion

DR is a common complication of DM in clinic, since the underlying mechanisms of DR pathogenesis are still not fully understood, there are still no effective treatments for DR in clinic. Therefore, uncovering the underlying mechanisms might help to cure DR in clinic. DR progression has been reported to be closely related with oxidative stress and inflammation [24], which could be targeted to alleviates DR development in rats [7]. Our clinical experiment results showed that MDA levels was increased, while SOD activity and GSH-PX activity were decreased in DR tissues comparing to the normal tissues, which suggested that oxidative stress was induced in DR tissues. HRECs has been widely used for the investigation of DR progression [25], hence we used high-glucose treated HERCs as the cell models in our experiments to explore the underlying mechanisms of DR pathogenesis in cellular levels. The cellular results were in accordance with the previous study [24] and our clinical results, which showed that high-glucose promotes oxidative stress, cell apoptosis and inflammation, inhibits cell viability. The results above indicated that oxidative stress and inflammation played important roles in DR progression, which might be served as a potential therapeutic agents for DR treatment.

*CKIP*-*1* has been reported to regulate multiple cell functions (cell proliferation [9, 10], apoptosis [11] and differentiation [26]) and involve in the development of several diseases including renal fibrosis [27], chronic heart failure [28], cancer [29] and so on. Recent studies also found that inflammation [12, 13] and oxidative stress [11] could be regulated by *CKIP*-*1*. For example, *CKIP*-*1* involved in the regulation of immune system and inflammation by regulating microglia M2 polarization [12] and macrophage M1/M2 polarization [13]. Besides, upregulation of *CKIP*-*1* alleviated oxidative stress in cultured hippocampal neurons [11]. However, there are still no literatures reporting the relationship between *CKIP*-*1* and DR pathogenesis. Based on the literatures above, it is reasonable to speculate that *CKIP*-*1* might participate in DR progression by regulating cell viability, oxidative stress and inflammation. The experiment results showed that *CKIP*-*1* is aberrantly downregulated in either DR tissues or high-glucose treated HRECs comparing to the Control group. Further results also showed that overexpressed *CKIP*-*1* reversed the effects of high-glucose treatment on HRECs viability, apoptosis, inflammation associated cytokines secretion and oxidative stress. Similarly, knock-down of *CKIP*-*1* had the opposite effects, which aggravated the effects of high-glucose on HRECs in terms of cell viability, apoptosis, inflammation and oxidative stress. Our cellular results validated our hypothesis and proved that *CKIP*-*1* played a protective role in DR pathogenesis.

Eventhough we have proved that high-glucose induced inhibition of HRECs viability, promotion of HRECs apoptosis, inflammation associated cytokines secretion and oxidative stress, the detailed mechanisms and downstream targets of *CKIP*-*1* are still unknown. *Nrf2*/*ARE* signaling is crucial for the regulation of oxidative stress in multiple diseases, such as neurological disorders [30], Parkinson diseases [31] and Alzheimer’s disease [32]. Of note, *Nrf2*/*ARE* signaling have been reported to be related with DM complications. For example, diabetic nephropathy (DN) could be ameliorated by activating *Nrf2*/*ARE* signaling pathway [33] and *Nrf2* has been deemed as a potential therapeutic approach to attenuate DR [34]. Our results showed that high-glucose treatment inhibited *Nrf2*/*ARE* activation in HRECs. In addition, knock-down of *Nrf2* enhanced the inhibiting effects of high-glucose on cell viability and the promoting effects of high-glucose on cell apoptosis, inflammation and oxidative stress. Our results indicated that activation of *Nrf2*/*ARE* signaling pathway might help to prevent DR progression, which was in compliance with the previous study [34]. Notably, previous studies have proved that *Nrf2*/*ARE* signaling pathway was the downstream target and could be activated by *CKIP*-*1* overexpression [11, 18, 27, 35]. However, it is still unclear whether *CKIP*-*1* alleviates DR development by regulating *Nrf2*/*ARE* signaling pathway. Our results showed that *CKIP*-*1* formed complex with *Nrf2* and activated *Nrf2*/*ARE* signaling pathway. Furthermore, the effects of overexpressed *CKIP*-*1* on high-glucose treated HRECs in terms of cell viability, oxidative stress, inflammation and apoptosis could be abrogated by synergistically knocking down *Nrf2*, which validated our speculation and indicated that *CKIP*-*1* participated in DR development by activating *Nrf2*/*ARE* signaling pathway.

## Conclusion

In conclusion, we proved that overexpressed *CKIP*-*1* could inhibit high-glucose induced HRECs apoptosis, inflammation as well as oxidative stress, and promote HRECs viability by regulating *Nrf2*/*ARE* signaling pathway. Our results will shed light on the discovery of new therapeutic agents for DR treatment in clinic.

## Materials and methods

### Clinical tissue samples

Human anterior lens capsule tissues were collected from DR patients (Fasting blood glucose = 7.99 ± 0.53, N = 20, DR group) and normal volunteers (Fasting blood glucose = 5.26 ± 0.47, N = 10, Control group) respectively from Zhejiang Provincial People’s Hospital. All the specimens were immediately refrigerated in liquid nitrogen and kept at − 80 °C for the following experiments. The average age of involved DR patients was 69.23 ± 7.08 years old and normal volunteers was 62.80 ± 7.80 years old, there was no statistical significance between the two group (*P *= 0.071). The gender (male/female) in the DR group and Control group were 11/9 and 6/4 (*P *= 0.794) respectively. All the clinical experiments were conducted in accordance with the principle of ‘Declaration of Helsinki’ [36] and got the approval from the ethics committee of Zhejiang Provincial People’s Hospital. Besides, the informed consent had been obtained from all the involved participants. The characteristics of the patients and normal volunteers have been shown on Table 1.

**Table 1:** Characteristics of the patient and the control group

	Control (n = 10)	Diabetic cataract (n = 20)	*P* value
Age (years)	66.70 ± 4.85	70.5 ± 5.31	0.068
Gender (male/female)	7/3	12/8	0.592
Fasting blood glucose (mmol/l)	5.35 ± 0.15	8.01 ± 0.67	< 0.001

### Vectors transfection

*CKIP*-*1* siRNAs were designed and synthesized by Shanghai GenePharma Co., Ltd (Shanghai, China), the sequences of the siRNAs were showed as follows, si-*CKIP*-*1* #1: 5′-CCGCUAUGUGGUGCUGAAA-3′; si-CKIP-1 #2: 5′-GGAACCAACCUCUUGUGCU-3′. Besides, the cDNA fragments of *CKIP*-*1* and pcDNA3.1 vectors were digested and prepared by EcoRV/XhoI double enzymes digestion. After that, the *CKIP*-*1* cDNA fragments were inserted into pcDNA3.1 vectors by T4 DNA ligase (Thermo Fisher Scientific, Waltham, MA, USA) to generate pcDNA3.1/*CKIP*-*1* overexpression vectors. Besides, siRNAs of *Nrf2* was constructed according to the previous study [37], the siRNA sequence of *Nrf2* was 5′-UAAUUGUCAACUACUGUCAGUU-3′. After the cell confluence reached approximate 80%, all the overexpressed vectors of siRNAs were transfected into HRECs by using Lipofectamine RNAiMAX Reagent (Invitrogen, Carlsbad, CA, USA) under the instructions of the manufacturer. After transfection, Real-Time qPCR and Western Blot were used to detect the efficiency of the above overexpressed vectors or siRNAs. After 24 h transfection, the culture medium was replaced with the medium containing high-glucose (25 mM) and co-cultured for 48 h.

### Cell culture and Cell counting kit-8 (CCK-8) assay

Human retinal endothelial cells (HRECs) were purchased from Angioproteomie company (Boston, MA, USA), which has been used in the previous study [38]. According to the company’s instruction, HRECs were cultured in endothelial cell medium (Sciencell, Carlsbad, CA, USA) supplemented with 5% fetal bovine serum (FBS), 100 μg/ml streptomycin (Wuhan Fortuna Chemical Co., Ltd, Wuhan, China). After transfecting cells with different vectors, cells were diluted to 1 × 10^4^ per well in the six-well plates and cultured in the incubator (Thermo Fisher Scientific, Waltham, MA, USA) under the temperature of 37 °C and a humidified atmosphere containing 5% CO_2_ and air. The cells were then exposed to 5.5 mM and 25 mM glucose for 48 h. Cell morphology was observed by inverted microscope (Olympus Corporation, Japan).

Cell viability was evaluated by CCK-8 assay according to the manufacturer’s instruction. In brief, HRECs were diluted and seeded in the 96-well plate in the concentration of 5000 cells per well. The CCK-8 solution (10 μl per well) was then added into the wells and incubated with the cells for 2 h in the standard culture conditions. Finally, the optical density (OD) values were detected and quantified in the wavelength of 450 nm to evaluate cell viability.

### Real-Time qPCR

The total RNA was extracted from DR tissues or high-glucose treated HRECs by Trizol reagent (Invitrogen corporation, Carlsbad, California, USA) according to the manufacturer’s instruction. The extracted total RNA was then reversely transcribed to complementary DNA (cDNA) by using TaqMan Reverse Transcription Reagents (Applied Biosystems, Foster City, CA, USA). The SYBR Green PCR Master Mix (Applied Biosystems, Thermo Fisher Scientific, Waltham, MA, USA) was employed to perform Real-Time qPCR to quantify the target cDNA by using the Applied Biosystems 7500 Real-Time qPCR System. The procedures were set as 95 °C for 10 min and 40 cycles of 95 °C for 15 s and 60 °C for 1 min according to the previous study. The primer sequences of the related genes are listed in the following Table 2. The relative mRNA expression levels were normalized to *β*-*actin* by using the 2^−ΔΔCt^ method.

**Table 2:** The primers of the involved genes

Gene	Primer sequences (strand)
*β*-*Actin*	Forward: 5′-CTCCATCCTGGCCTCGCTGT-3′ Reverse: 5′-GCTGCTACCTTCACCGTTCC-3′
*CKIP*-*1*	Forward: 5′-AATTCTGCGGGAAAGGGATTT-3′ Reverse: 5′-AACACCTCCTGACTGTTTTTCTC-3′
*Nrf2*	Forward: 5′-GACCTAAAGCACAGCCAACACAT-3′ Reverse: 5′-CTCAATCGGCTTGAATGTTTGTC-3′

### Western Blot

The total proteins in the clinical tissues and HRECs were extracted by using RIPA lysis buffer (Beyotime Biotechnology, Shanghai, China) according to the manufacturer’s instruction. The nuclear fractions were extracted by using the Nuclear/Cytosol Fractionation Kit (BioVision, Inc., Milpitas, CA, USA) according to the previous study [39]. The lysates were obtained by centrifugation and protein concentrations were quantified by using a BCA kit (Beyotime Biotechnology, Shanghai, China). The 10% SDS–polyacrylamide gel was used to separate the target proteins, which were then transferred to PVDF membranes (Millipore, Bedford, MA, USA). After blocking the membranes with 5% skim milk diluted in TBST (Tris-buffered saline containing 0.1% Tween-20) for 45 min at 37 °C, the membranes were then incubated with the primary antibodies against *β*-*actin* (1:2000, #ab8226, Abcam, UK), *CKIP*-*1* (1:1000, #ab91489, Abcam, UK), *Nrf2* (1:1000, #ab137550, Abcam, UK), *Bcl*-*2* (1:1000, #ab32124, Abcam, UK), *cleaved Caspase 3* (1:1000, #ab13847, Abcam, UK), *Bax* (1:1000, #ab53154, Abcam, UK), *Lamin B* (1:500, #ab133741, Abcam, UK), *Tublin* (1:2000, #ab18251, Abcam, UK), *HO*-*1* (1:1000, #ab13248, Abcam, UK), *NQO*-*1* (1:1000, #ab80588, Abcam, UK), *γGCS* (1:1000, #ab59956, Abcam, UK) and *SOD* (1:1000, #ab80946, Abcam, UK) at 4 °C overnight. The membranes were next washed with TBST three times and incubated with horseradish peroxidase (HRP)-conjugated secondary antibody (Abcam) for 1 h at room temperature. After that, ECL Western Blotting Detection Kit (GE Healthcare Bio-Science, Pittsburgh, PA, USA) was next purchased to detect the protein bands, the optical density of the protein bands were quantified by Image J software and normalized to β-actin.

### Detection of oxidative stress by MDA assay kit, SOD kit and GSH-Px assay kit

Malondialdehyde (MDA) was the degraded product of polyunsaturated lipids, which could be used to measure the level of lipid peroxidation and oxidative stress. MDA could react with thiobarbituric acid as a thiobarbituric acid reactive substances (TBARS) to form a 1:2 MDA-TBA adduct, hence measuring the contents of TBARS reflected MDA levels. The MDA assay kit (Beyotime Biotechnology, Shanghai, China) was used to evaluate TBARS according to the manufacturer’s instruction.

Superoxide dismutase (SOD) played an important role in the regulation of oxidative stress, which could help to eliminate reactive oxygen species (ROS) and protect cells from the damage of oxidative stress. Hence, SOD activity could be used as a biomarker to evaluate oxidative stress in cells. The SOD assay kit (Nanjing Jiancheng Bioengineering Institute, Nanjing, China) was used to detect SOD activity in high-glucose treated HRECs according to the manufacturer’s instruction.

Glutathione peroxidase (GSH-PX) was an important antioxidant enzyme in cells, which could be used to evaluate the levels of oxidative stress. The GSH-PX assay kit (Nanjing Jiancheng Bioengineering Institute, Nanjing, China) was used to estimate GSH-PX activity in high-glucose treated HRECs according to the manufacturer’s instruction. The absorbance of the samples was determined at 412 nm for GSH-PX at the end of reaction on a microplate reader.

### Detection of cell apoptosis by Calcein-AM/PI double stain kit

Calcein-AM/propidium iodide (PI) double stain kit (Shanghai Yeasen Corporation, Shanghai, China) was used to detect cell apoptosis according to the manufacturer’s instruction. In brief, high-glucose treated HRECs were washed with assay buffer for 3 times, and cells were suspended with assay buffer at the density of 1 × 10^5^/ml. After that, the working reagent containing Calcein-AM and PI at the ratio of 4:3 was added and incubated with the cells in 37 °C for 15 min. The inverted fluorescence microscope (Olympus Corporation, Japan) was employed to observe HRECs apoptosis ratio.

### Enzyme-linked ImmunoSorbent Assay (ELISA)

The inflammation associated cytokines (TNF-α, IL-6 and IL-1β) in the experiments were detected by TNF-α ELISA Kit (#ab181421, Abcam, UK),IL-6 ELISA Kit (#ab46027, Abcam, UK) and IL-1β ELISA Kit (#ab46052, Abcam, UK) respectively. In brief, the culture supernants in the Control group and high-glucose treated group were collected. The expression levels of TNF-α, IL-6 and IL-1β were detected according to the manufacturer’s instruction.

### Co-immunoprecipitation (CO-IP)

The Flag-labeled *CKIP*-*1* (Flag-*CKIP*-*1*) and HA-labeled *Nrf2* (HA-*Nrf2*) plasmids were used in the CO-IP experiment. The Flag-*CKIP*-*1* plasmid or HA-*Nrf2* plasmid was transfected into the HRECs by using the Lipofectamine RNAiMAX Reagent (Invitrogen, Carlsbad, CA, USA) according to the manufacturer’s protocol. After vectors transfection for 24 h, the total proteins in HRECs were extracted by using RIPA lysis buffer (Beyotime Biotechnology, Shanghai, China) according to the manufacturer’s instruction. The supernatant was collected and incubated with protein A-Sepharose (Sigma, USA) at 4 °C for 1 h. The following sodium dodecyl sulfate polyacrylamide gel electrophoresis (SDS-PAGE) was next used to separate the target proteins. After that, the anti-Flag (1:1000, Sigma, USA) and anti-HA (1:1000, Sigma, USA) were used as the primary antibodies for further immunodetection. Besides, the peroxidase-conjugated anti-rabbit *immunoglobulin G* (*IgG*) (1:2000, #ab133470, Abcam, UK) was used as the secondary antibody. ECL Western Blotting Detection Kit (GE Healthcare Bio-Science, Pittsburgh, PA, USA) was next used to quantify the protein contents.

### Luciferase reporter gene system

The ARE promoter fragment was subcloned into pGL3 basic vector to produce pGL3-ARE luciferase vectors, which were then transfected into HRECs by using the Lipofectamine RNAiMAX Reagent (Invitrogen, Carlsbad, CA, USA). The HRECs were then treated with low-glucose (5.5 mM), high-glucose (25 mM), high-glucose and pcDNA3.1-NC vectors, high-glucose and pcDNA3.1-*CKIP*-*1* vectors respectively. The pRL-TK vector was next used to normalized the transfection efficiency. After that, the HRECs were lysed by RIPA lysis buffer (Beyotime Biotechnology, Shanghai, China) for further analysis. The dual-luciferase reporter assay system (Promega, Madison, Wis., USA) was used to measure luciferase activity according to the manufacturer’s protocol.

### Statistical analysis

All the experiments were conducted at least for three times. The data was collected and showed as mean ± standard deviation (SD) for further analysis. Student’s t test was used to compare the two groups in the experiments. One way analysis of variance (ANOVA) was used to compare multiple groups (more than 3 groups) in the experiments. Statistical analysis was performed using the SPSS 18.0 software. Differences of P < 0.05 were considered as statistical significance.

## Supplementary information

**Additional file 1: Figure S1.** The effects of high-glucose treatment on cell viability and inflammation. (A, C) Cell viability was detected by using CCK-8 assay. (B) The inflammatory cytokines (TNF-α, IL-6 and IL-1β) were detected by using an ELISA kit. The data above in one experiments were repeated at least 3 times and performed as mean ± standard deviation (SD), *P < 0.05, **P < 0.01 and ***P < 0.001.

**Additional file 1: Figure S2.** The schematic model of this study.

## Data Availability

All data generated or analyzed during this study are included in this published article (Additional file 2).

## Abbreviations

*MDA*: malondialdehyde
*SOD*: superoxide dismutase
*GSH*-*Px*: glutathione peroxidase
ELISA: Enzyme-linked ImmunoSorbent Assay
CO-IP: co-immunoprecipitation
DR: diabetic retinopathy
DM: diabetes mellitus
*CKIP*-*1*: casein kinase 2 interacting protein-1
*Nrf2*: nuclear factor E2-related factor 2
*ARE*: antioxidant response element
*ICAM*-*1*: intercellular cell adhesion molecule-1
