# Supplementary figures and images for: Upregulation of CKIP-1 inhibits high-glucose induced inflammation and oxidative stress in HRECs and attenuates diabetic retinopathy by modulating Nrf2/ARE signaling pathway: an in vitro study
Source: Cell Biosci. 2019 Aug 23;9:67. doi: 10.1186/s13578-019-0331-x (PMC6708125; doi:10.1186/s13578-019-0331-x)

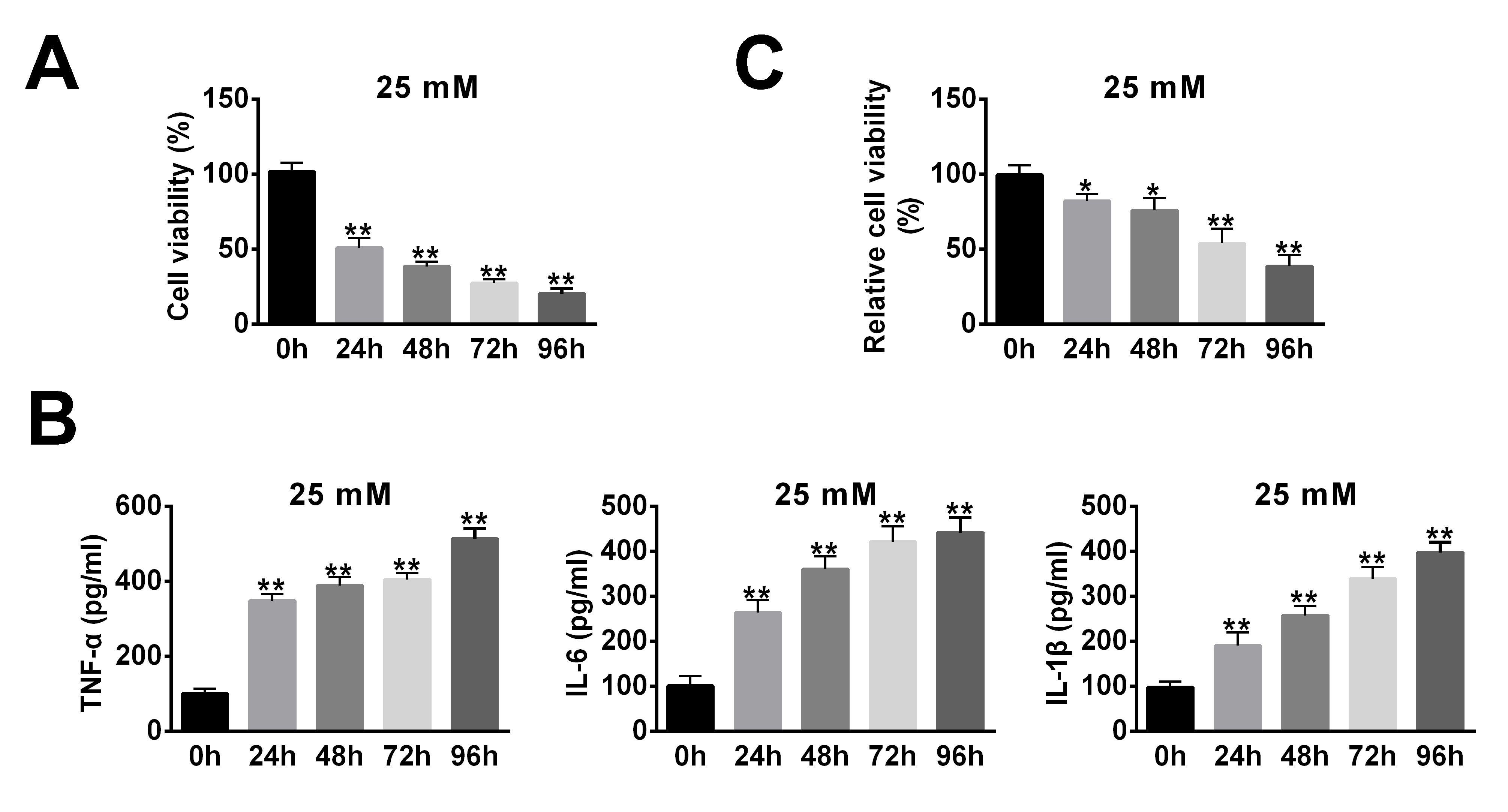

Supplement: Supplementary file 1 — Additional file 1: Figure S1. The effects of high-glucose treatment on cell viability and inflammation. (A, C) Cell viability was detected by using CCK-8 assay. (B) The inflammatory cytokines (TNF-α, IL-6 and IL-1β) were detected by using an ELISA kit. The data above in one experiments were repeated at least 3 times and performed as mean ± standard deviation (SD), *P < 0.05, **P < 0.01 and ***P < 0.001. [file 13578_2019_331_MOESM1_ESM.jpg]

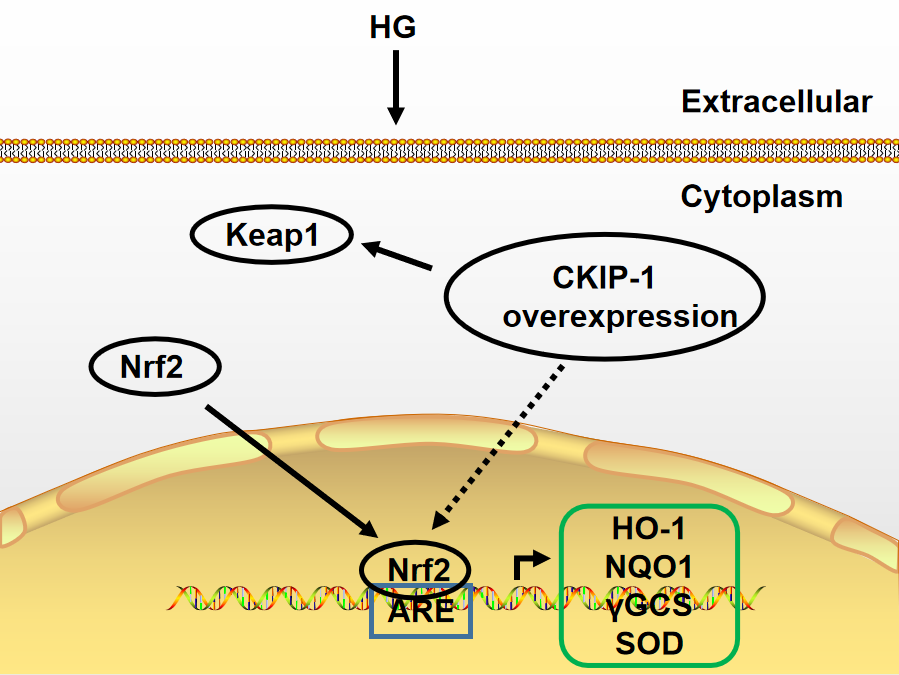

Supplement: Supplementary file 2 — Additional file 1: Figure S2. The schematic model of this study. [file 13578_2019_331_MOESM2_ESM.tif]
